# Integrative assessment of climate change for fast-growing urban areas: Measurement and recommendations for future research

**DOI:** 10.1371/journal.pone.0189451

**Published:** 2017-12-12

**Authors:** Sebastian Scheuer, Dagmar Haase, Martin Volk

**Affiliations:** 1 Humboldt-Universität zu Berlin, Geography Department, Landscape Ecology Lab, Berlin, Germany; 2 Helmholtz-Centre for Environmental Research Leipzig—UFZ, Department of Computational Landscape Ecology, Leipzig, Germany; Universidade de Vigo, SPAIN

## Abstract

Over the 20^th^ century, urbanization has substantially shaped the surface of Earth. With population rapidly shifting from rural locations towards the cities, urban areas have dramatically expanded on a global scale and represent crystallization points of social, cultural and economic assets and activities. This trend is estimated to persist for the next decades, and particularly the developing countries are expected to face rapid urban growth. The management of this growth will require good governance strategies and planning. By threatening the livelihoods, assets and health as foundations of human activities, another major global change contributor, climate change, became an equally important concern of stakeholders. Based on the climate trends observed over the 20^th^ century, and a spatially explicit model of urbanization, this paper investigates the impacts of climate change in relation to different stages of development of urban areas, thus evolving a more integrated perspective on both processes. As a result, an integrative measure of climate change trends and impacts is proposed and estimated for urban areas worldwide. We show that those areas facing major urban growth are to a large extent also hotspots of climate change. Since most of these hotspots are located in the Global South, we emphasize the need for stakeholders to co-manage both drivers of global change. The presented integrative perspective is seen as a starting point to foster such co-management, and furthermore as a means to facilitate communication and knowledge exchange on climate change impacts.

## Introduction

Rapid urbanization is a key driver of global change, and a significant contributor to climate change [1]. Today, cities are already home to more than half of the world's population and focal points of economic and financial activities, as well as hubs of innovation. Built-up urban areas are expanding dramatically, and at exceptional rates, demanding increasingly more land and natural resources. The total global urban population is estimated to double by 2050, when compared to 2010 [2,3]. As exemplified most recently when the city of Houston was hit by hurricane Harvey, cities are particularly prone to natural hazards due to their high populations and the high concentration of a wide range of exposed assets. Cities are also prone to the impacts of climate change and climate change-related hazards, which may considerably affect human health and well-being.

Of these various hazards, water scarcity, drought, flooding and heatwaves are considered the primary climate change-related threats for the urban space [4,5]. Water scarcity and drought can be considered as long-term stresses, that are likely to be exacerbated by climate change. Both conditions can already frequently be observed in many cities, e.g., in the large urban agglomerations of California and Arizona [6,7]. On the contrary, short-term shocks, such as extreme weather events, can be shown to be further exacerbated by the unique characteristics of urban space [5,8]. For example, the large areas of ground that are impervious to groundwater penetration in cities and their hinterland may contribute to flooding after heavy precipitations events. Cities in low-lying, coastal areas are also at risk of coastal flooding. In addition, extreme temperatures during heat spells or heatwaves may be further exacerbated by urban heat islands [8–11]. Climate change may thus exacerbate both hydrological risks—i.e., risk of riverine and coastal flooding—as well as climatological/meteorological risks such as the risk of excess temperatures [12]. In this context, it needs to be noted that this paper considers the aforementioned shocks and stresses, i.e., types of natural hazards, as manifestations of climate change, or as the impacts of climate change, respectively, and these are in turn conceptualized as observed trends in various selected indicators.

There is an increasing need to estimate the magnitude and impact of climate change on cities and the subsequent derivation of risk management and climate change adaptation options because of the increasing shift of urbanization towards the Global South. Today, more than one third of the world's total population already lives in urban areas of low- and middle-income developing countries [13], which are expected to absorb the majority of urban growth within the next decades [3,14]. However, it is also these developing regions that are most often especially vulnerable to climate change due to factors such as their high proportion of poorer communities and lack of proper governance [3,13,15].

Here, vulnerability is defined as a system’s susceptibility or inability to cope with the adverse effects of climate change [16]. Thus, vulnerability analysis focuses on the assessment of sensitivity and capacity for adaptation to specific climate change-related hazards, where sensitivity and adaptation capacity of the anthropogenic system are often measured using comprehensive sets of biophysical and socioeconomic indicators [17–19]. Due to both the high need for adaptation in cities, as outlined above, as well as the increasing urban commitment to action on climate change, the assessment of climate change impacts and vulnerabilities is increasingly shifting towards the urban space [8,20].

Estimating climate change impacts, sensitivity and adaptive capacity further allows the identification of gaps in current adaptation efforts, i.e., impact-specific adaptation options not yet covered in urban adaptation strategies [5]. The identification of the need for, and gaps in adaptation allows actions to be prioritized for an effective allocation of limited financial resources. Furthermore, such an identification can aid in focusing available resources on areas where the need for action is highest. However, it has been found that in 2014 out of 401 urban agglomerations worldwide with more than one million inhabitants, 81% provided no evidence of any kind of adaptation to climate change [9]. This included most of the Eastern European, African, Central American, Central and South-Asian cities that were studied. In contrast to this, in 2016, more than 6,700 European cities are in the process of committing to mitigation or are in an early state of assessing vulnerabilities to climate change. Only a few cities, most notably Barcelona, Rotterdam, Copenhagen and Helsinki, have advanced to the point of exploring monitoring schemes for actions that have already been put into action [4].

Determining adaptation needs and identifying adaptation gaps relies heavily on knowledge about the magnitude of climate change at local level. Unfortunately, local knowledge is a heavily fragmented body of knowledge [21]. This fragmentation—in combination with a lack of scientific research and evidence at local levels, as well as a lack of public awareness, funding, and cooperation—poses an important barrier to adaptation [22].

In particular, the lack of scientific knowledge at local levels needs to be addressed. Consequently, in this paper, urban hotspots of climate change are identified, i.e., urban land where climate change will likely have a comparatively high impact. In so doing, land and water managers, mayors and risk management shall be provided with a common view of climate change impacts in order to facilitate the transfer of knowledge, stimulate a wider stakeholder discussion on the scope and magnitude of required adaptation measures, and subsequently enable adaptation strategies to resonate between distal urban locations [23]. It has been shown that such an exchange of scientific knowledge and international expertise on climate change adaptation is urgently needed by decision-makers [22]. Moreover, the approach presented here will provide anchor points for more specific future research and may also serve as an additional indicator for the discovery of adaptation gaps.

To establish the aforementioned common perspective, this paper presents an integrative assessment in the form of an observation of changes over the 20^th^ century at a global level, however, it looks at urban land on a local scale. This approach assesses climate change impacts as trends or changes, respectively, in mean annual surface air temperature and mean annual precipitation, which in turn are seen as possible drivers of long-term stresses. These observed trends will subsequently be used to derive an integrative, quantitative measure of climate change. In so doing, a quantitative frame of reference for the magnitude of climate change impacts at the local level is elaborated for the global urban land, and hotspots, which are identified accordingly. These long-term trends are further contrasted with selected indicators of heat as an example for the determination of likely trends in short-term shocks.

The assessed trends and proposed measure of climate change “magnitude” will additionally be put into context with a spatially explicit model of urbanization, that elicits different states of urban development. These states include, e.g., rapidly developing, comparatively young cities that are characterized by extensive spatial extension and large population growth as well as relatively old cities that are spatially and demographically stagnating [14]. In line with the literature [24,25], this model of urbanization also shows that hotspots of urbanization are predominantly located in the Global South [26]. In these developing countries, it is more likely that governance performs poorly due to factors such as a lack of efficiency, lack of economic or democratic freedom and widespread corruption. Thus, these countries are not able to meet the demands of sustainable planning and risk management [27]. In this paper, as outlined above, it will be shown that these cities may also be hotspots of climate change where the demand for scientific knowledge to support adaptation is high. In so doing, we make a case for the need to co-manage urbanization and climate change.

The remainder of the paper is organized as follows: The following section proposes an integrative measure of climate change and presents the materials and methods used. Furthermore, attention is given to the aforementioned spatially explicit model of urbanization [14], from which the most important findings that are relevant to this paper will be summarized. Results are presented in a step-by-step manner in section 3 in which firstly, the observed changes and trends in climatic parameters over the 20^th^ century are summarized. Secondly, the proposed integrative perspective on climate change is developed, and subsequently exemplified by selected case cities across the globe. Finally, these findings are put into the context of urban development, i.e., the assessed magnitude of climate change observed over the 20^th^ century will be aggregated on the level of hypothesized stages of urban development. Thereby, attention will be guided towards specific hotspots of urbanization and climate change. The paper closes with a discussion and conclusions.

## Material and methods

### Material

#### Climate data

To determine long-time temperature trends, this study makes use of gridded monthly mean terrestrial surface air temperature data (in °C) for the years 1901–2014 [28–30]. Long-term trends in the total monthly precipitation (in mm) are assessed using the Global Precipitation Climatology Centre (GPCC) Full Data Reanalysis dataset for the years 1901–2013 [31,32]. Both datasets have been chosen due to the available length of the time series, and their comparatively high spatial resolution of 1/2° x 1/2°, which is identical for both datasets. This high spatial resolution makes both datasets better suited for the scope of this case study when compared to datasets with a coarser resolution, and also facilitates the integration of both datasets, e.g., by avoiding resampling of grid values. Moreover, both datasets are also well suited for this study due to their global coverage and completeness, thus avoiding missing data.

The subsequent determination of heat indicators is based on the daily maximum temperature, which has been derived from the NOAA-CIRES 20^th^ Century Reanalysis version 2 dataset, which provides 4 times daily temperature estimates for the years 1871–2012 as a Gaussian grid with a spatial resolution of approximately 2° by 2° [33–35], again covering the whole terrestrial surface.

#### Urban land-use data

A gridded urban land-use dataset with a resolution of 1/6° x 1/6° has been derived on a global scale from the HYDE 3.1 land-use database [36,37] to approximate the age, total spatial extent, and variability of the spatial extent of urban areas for the period 1900–2000. A spatially explicit model of urban development has been proposed on the basis of these indicators [14]. From this model, the concept of “maturity” of urban land is of interest for the case made in this paper. Maturity can be understood as the relationship between the age of urban land and its spatial and demographic “stability”. It is conceptualized using local indicators of spatial association (LISA), i.e., four clusters representing spatial autocorrelation [14]. Matured cities are considered as stable, i.e., lacking substantial large-scale urban growth; this refers to both their spatial extent and their population, either growing relatively slowly or being in stagnation or decline [14]. Many European core cities fall in this category. Less matured cities, on the other hand, could be referred to as unstable, i.e., still experiencing (rapid) population growth in conjunction with a high variability of their spatial extent due to often fast-paced and tremendous spatial expansion. Cities in developing countries are typical examples of this category.

In this context, it needs to be noted that the terms urban land, urban area, city, or urban space are used interchangeably in this paper. This is primarily a consequence of the scale of analysis. Due to the global-scale approach, individual cities may be coarsely delineated compared to using e.g. administrative data. Furthermore, since the underlying data source is gridded, the paper refers to grid cells which are classified as urban, thus containing urban area of a given extent.

### Methods

#### Integrative measure of the magnitude of climate change

As previously described, this paper proposes an integrative, quantitative measure of the overall “magnitude” of climate change at local level to subsume the observed long-term trends in mean annual surface air temperature and mean annual total precipitation for the years 1901–2000. We propose a quantification of this measure of magnitude in the form of the Euclidian distance $\vec{d}$, which is the greater the higher the change observed in either long-term trend. Since $\vec{d}$ is measured in the units of the inputs used, both long-term trends need to be of the same unit. For that reason, relative trends, i.e., dimensionless values, will be used for assessing the Euclidian distance $\vec{d}$. Hence, in the following, firstly, absolute long-term temperature and precipitation trends will be assessed. Secondly, based on these absolute trends, the relative trends will be estimated. These relative trends are subsequently used for the determination of the Euclidian distance $\vec{d}$ as integrative measure of climate change magnitude. Finally, heat indicators are assessed and put into the context of the aforementioned long-term trends (S1 Fig).

#### Determination of absolute long-term trends

For the estimation of long-term trends (changes) in the climate parameters surface air temperature and precipitation, the mean monthly terrestrial surface air temperature [28–30] and the monthly total precipitation [31,32] have firstly been aggregated to obtain the gridded mean annual surface air temperature (°C) and mean annual total precipitation (mm), respectively, for each grid cell, and for each year of the analysis period 1901–2000 (S1 Fig).

The Theil-Sen estimate is then used to assess the long-term trends over the analysis period, per grid cell. The Theil-Sen estimate is a linear trend measure that is relatively robust against non-normal data and outliers, where the change (trend) in a time series is expressed as the median slope *b* obtained from all possible combinations of two values in the time series [38,39]. To estimate this median slope *b*, the time series 1901–2000 derived for each grid cell has been pre-whitened using Zhang's method [38] and de-autocorrelated; missing data values were not present in the data. Subsequently, the long-term trends in the form of the median slope *b* have been determined [39], as implemented in the R package "zyp" [40]. A non-parametric Mann-Kendall (MK) test for the absence (H_0_) or presence (H_A_) of a significant monotonic (linear or non-linear) trend was conducted simultaneously. This allows identifying significant trends. Similar to the Theil-Sen estimate, the MK test procedure is quite robust against (non-normal) data with outliers. Both methods are commonly used for analysing hydro-meteorological time series [39,41–47].

#### Determination of relative trends

Absolute trends might not be easily interpretable for any given location, and particularly hamper comparisons between different locations on Earth due to the differences in the observed absolute values. For that reason, relative trends will be used to facilitate comparisons and trend interpretations between different locations. As outlined above, these relative trends will furthermore be used to determine the proposed integrative measure of the “magnitude” of climate change.

To obtain relative changes (trends), an early 20^th^ century baseline has firstly been assessed by estimating the mean annual surface air temperature and the mean annual total precipitation for the period 1901–1931. The observed absolute long-term trends 1901–2000 have then been normalized in regard to this baseline *p*_*b*_ (S1 Fig). In so doing, the relative change *d* has been determined for each grid cell, with *d* = *b*/|*p*_*b*_|, where *b* is the absolute change expressed in the form of the Theil-Sen slope as introduced above, and *p*_*b*_ is equal to the mean annual surface air temperature, or the mean annual total precipitation, respectively, of the baseline period 1901–1931.

#### Determination of the Euclidian distance as integrative measure of climate change

In a next step, the bivariate distribution of the estimated relative trends is mapped to the global urban land, i.e., grid cells defined as urban [14]. In so doing, for each corresponding grid cell, the proposed Euclidian distance $\vec{d}$ is being assessed, with

$$\vec{d}=\sqrt{d_{air}^{2}+d_{prc}^{2}}$$ (1)

where *d*_*air*_ is equal to the estimated relative change in mean annual surface air temperature in the given grid cell, and *d*_*prc*_ is equal to the observed relative trend in mean annual total precipitation, respectively. As mentioned earlier, the Euclidian distance $\vec{d}$ will be the greater the higher *d*_*air*_ and/or *d*_*prc*_. It will thus help to identify urban hotspots of climate change.

#### Determination of trends in heat indicators

To identify changes in heat patterns, the number of heat days and heat spells has been determined as possible indicators on an annual basis for the analysis period 1901–2000 as follows (S1 Fig): (i) For each day of the year, the maximum daily temperatures *t*_*max*_ of a 31-day window centred on the observed day of the year have been determined for a 30-year reference period (1970–2000); (ii) Subsequently, the threshold temperature for a heat day *t*_*crit*_ has been derived for each day of the year as the 90% quantile of this reference set of maximum daily temperatures; (iii) For each individual day in the period 1901–2000, it has been determined if the day in question constitutes a heat day. That is the case if the observed daily maximum temperature *t*_*max*_ > *t*_*crit*_ [48].

Consequently, the total number of (non-consecutive) heat days per year *h*_*d*_ for the complete analysis period 1901–2000 has been elicited. In addition, the mean temperature of all heat days per year *t*_*h*_ has been derived as well as the total number of heat spells *w*_*h*_ and their average duration *w*_*d*_ (in days). Here, a heat spell is defined as a period of at least 3 consecutive heat days [48]. Finally, the observed trends in each of these heat indicators over the analysis period 1901–2000, as well as their significance, have again been assessed using the Theil-Sen method and MK tests [39].

## Results

### Absolute long-term trends in mean annual surface air temperature and mean annual total precipitation

**Fig 1:**
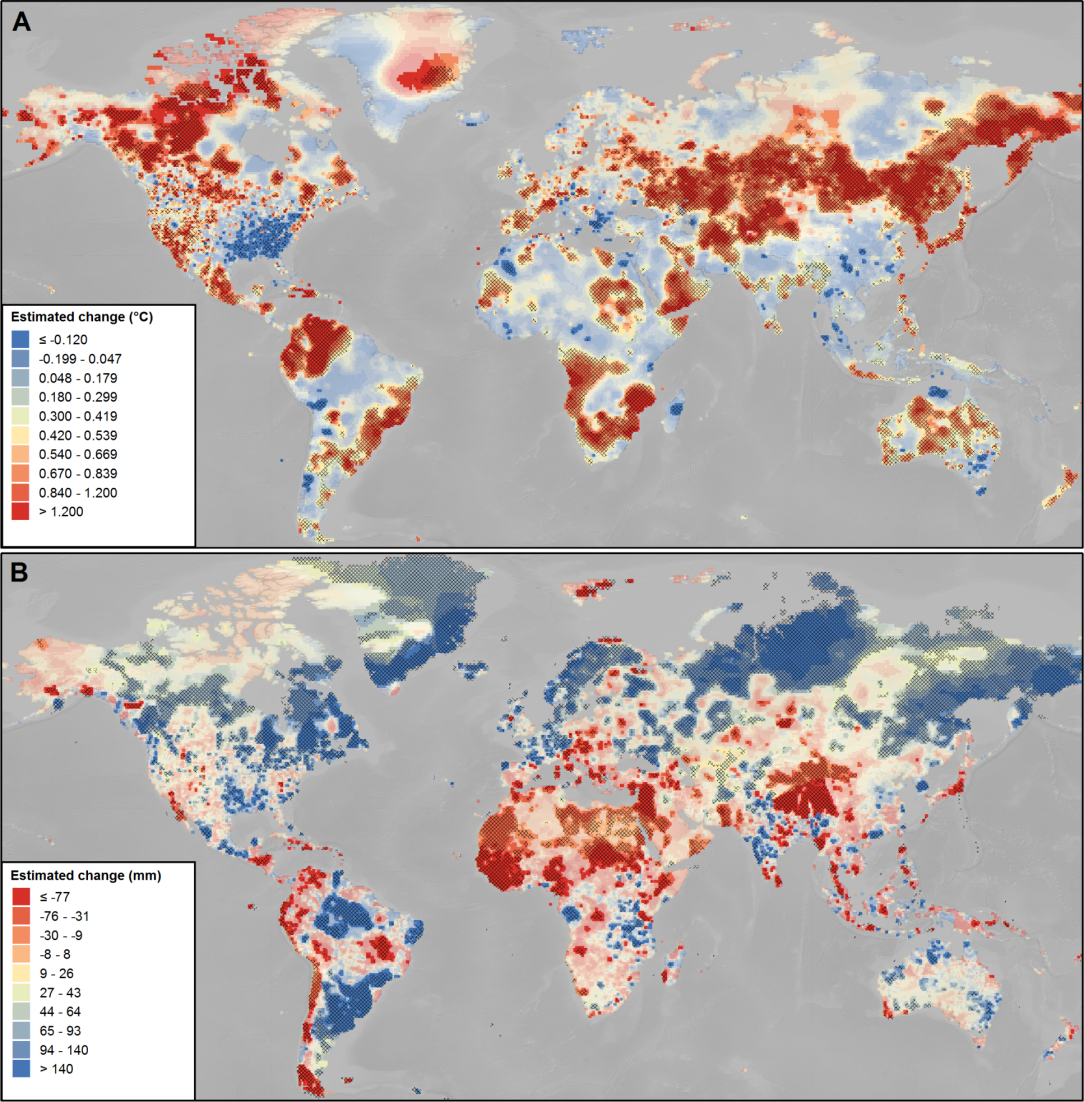
Absolute long-term trends 1901–2000 in mean annual surface air temperature and mean annual total precipitation, estimated by Sen's slope *b*. (A) Trend in mean annual surface air temperature (°C). (B) Trend in mean annual total precipitation (mm). Non-significant trends are shown in translucent colours. Opaque colours indicate trends that are significant at the 10% level. Trends that are significant also at the 5% level are indicated by crosshatching. The classification corresponds to deciles.

Fig 1 shows the absolute trends in mean annual surface air temperature and mean annual total precipitation over the 20^th^ century based on Sen's slope *b*. In the map, and similar to IPCC [49], significant trends are highlighted at the 10% and at the 5% level; in the remainder of the paper, trends will be considered significant when a significance level of at least 10% is reached. This corresponds to very likely trends according to IPCC [49].

Significant trends in mean surface air temperature imply warming in most cases (Fig 1A). In accordance with IPCC, particularly strong warming is observed in the higher latitudes, with temperature increases exceeding 4.5°C for parts of the arctic region [50]. A comparatively high increase in mean surface air temperature has also been estimated along a band stretching from the Baltics to the Russian Pacific coast. Cooling is estimated substantially less frequent, and is mainly observed in the South-Eastern United States, the Southern Balkan and small patches across Africa and the Middle East, China, South-East Asia and Australia [51]. Concerning precipitation, a 20^th^ century increase for the mid-to high latitudes, and decreases in precipitation particularly in subtropical areas have been described [51]. Findings shown in Fig 1B depict this general pattern, with a significant decline of precipitation in Northern (Saharan) Africa, the Mediterranean, the Middle East, Latin America and across the Andes, the Balkan, as well as Central and South-East Asia.

### Relative long-term trends in mean annual surface air temperature and mean annual total precipitation

**Fig 2:**
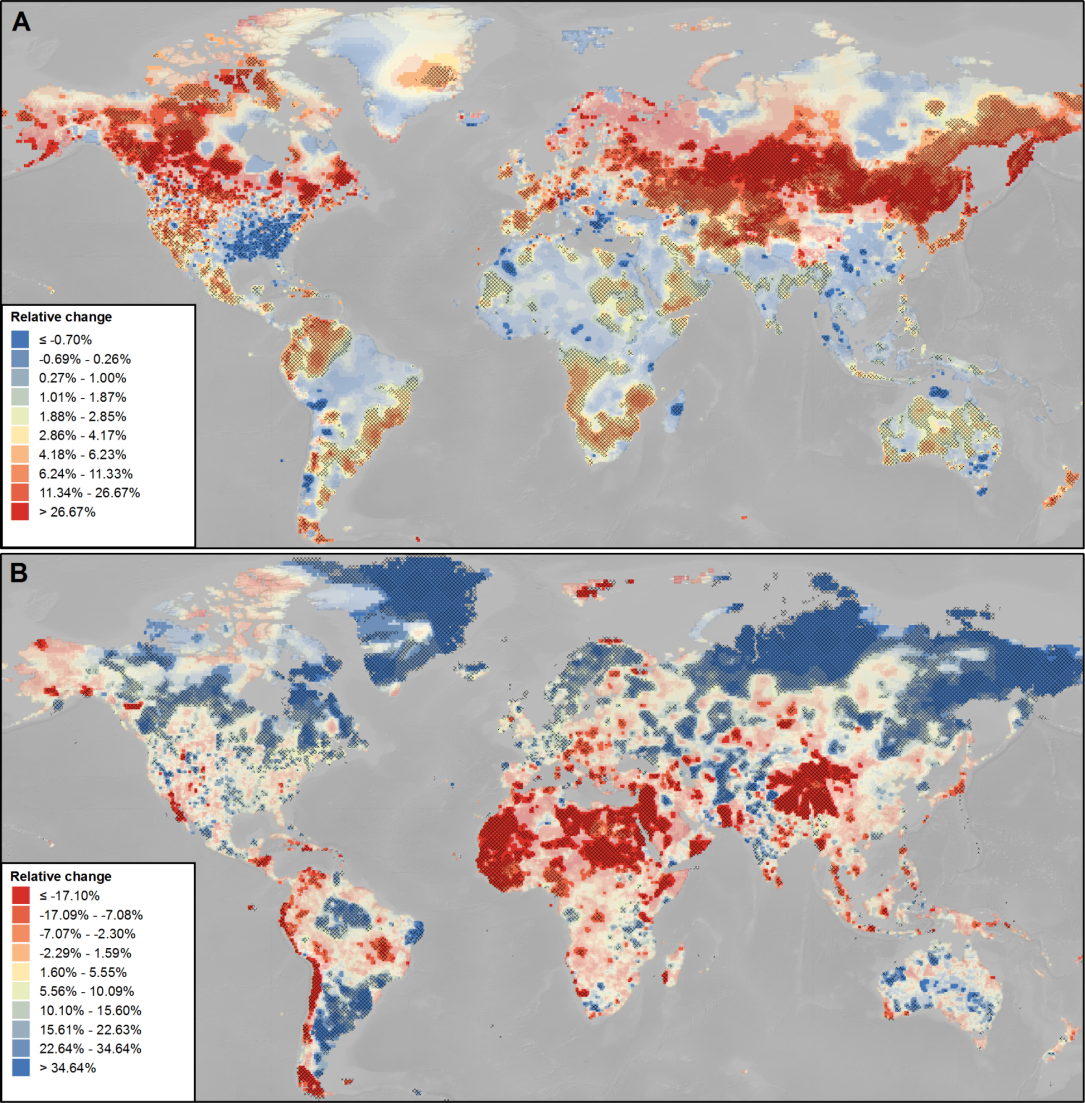
Long-term trends 1901–2000 in mean annual surface air temperature and mean annual total precipitation relative to the 1901–1931 baseline. (A) Relative change in mean annual surface air temperature (%). (B) Relative change in mean annual total precipitation (%). The indication of the significance of trend is according to Fig 1. The classification corresponds to deciles.

The relative change per grid cell, i.e., the estimated 20^th^ century trend relative to the 1901–1931 baseline, is shown in Fig 2. The general patterns here match the estimated absolute trends depicted in Fig 1. However, it becomes clear that cooling, in regard to the baseline, is only of small magnitude, whilst warming is considerably stronger, with highest increases equating to as much as 26% and higher. It is also interesting to note that increases of surface air temperature in Fennoscandia and North-Western Russia were mostly not significant at the 10% level. However, the corresponding relative changes are notable despite the non-significance of the modelled trends. Conversely, the significant increases in temperature that were modelled for Southern Africa and Central America, estimated on the higher end of the absolute scale, correspond to comparatively small increases in relative terms. Regarding precipitation, particularly the extreme increases—e.g., Northern and North-Western Russia and Southern Brazil—and decreases—e.g., North Africa and the Chinese Tibetan Plateau—correspond to the highest changes in both absolute as well as relative terms.

**Fig 3:**
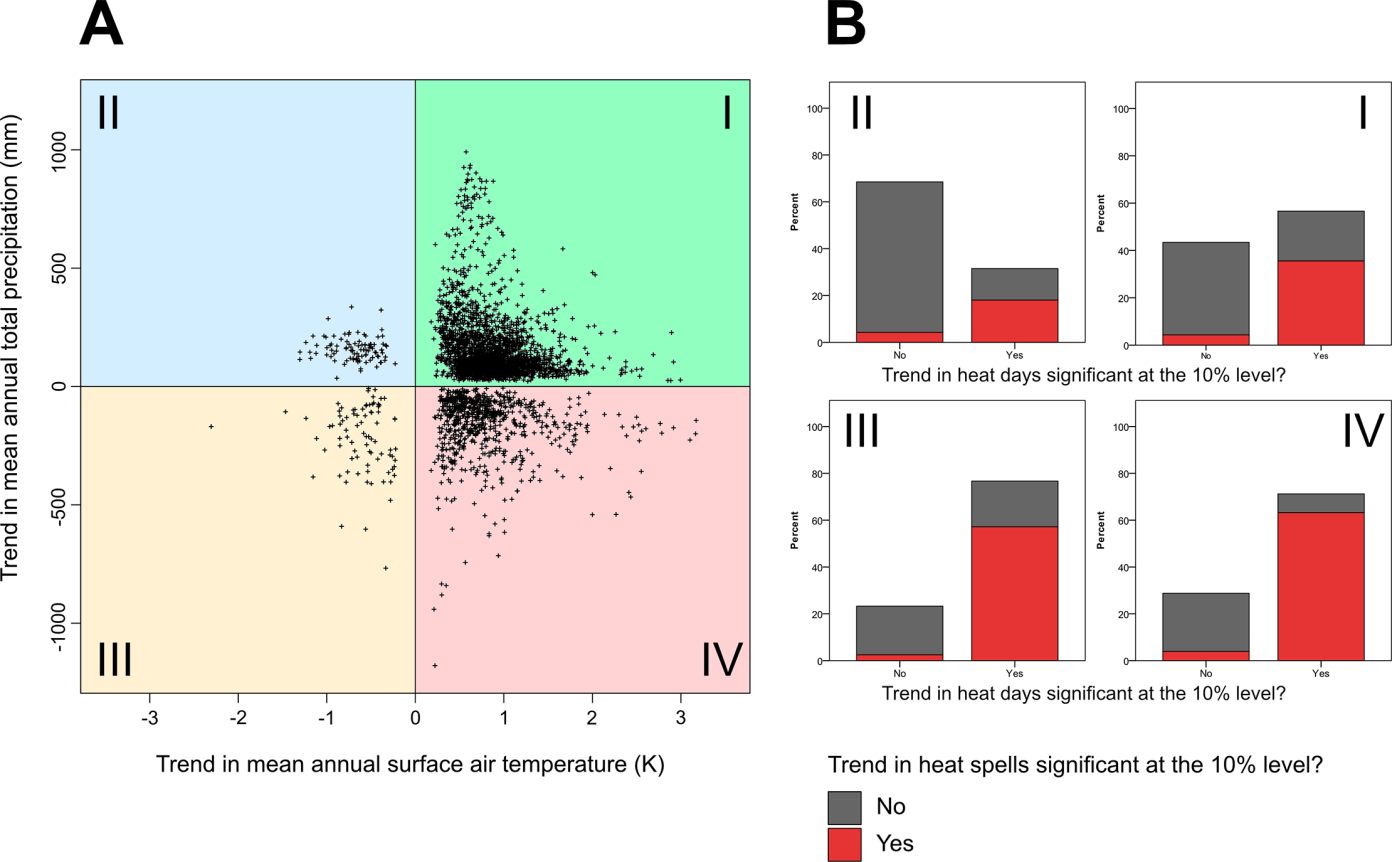
Absolute long-term trends 1901–2000 in mean annual surface air temperature and mean annual total precipitation for the global urban land (per grid cell) and the combined share of non-significant as well as significant trends in heat parameters. (A) Combined trends in mean annual surface air temperature (K) and mean annual total precipitation (mm) that are significant at the 10% level. The resulting direction of change corresponds to the coloured quadrants labelled I to IV. Generally, warming could be regarded as the dominating trend over the 20^th^ century for urban land. However, if seen in conjunction with precipitation, two major directions of change become apparent: Warming combined with increasing rainfall (quadrant I shown in green), or with a simultaneous decrease in precipitation (quadrant IV shown in red). (B) Share of (non-)significant trends in (non-consecutive) heat days per year in conjunction with the combined share of (non-)significant trends in the total number of heat spells per year (as colour coding of each column), per direction (quadrant) of change.

It can be concluded that, in principle, urban adaptation to climate change requires dealing with one out of four distinct directions of change. These directions of change are indicated by the quadrants depicted in Fig 3A. This figure shows the combined trends in both climatic parameters significant at least at the 10% level for the global urban land. The green quadrant I characterizes a positive trend in both mean surface air temperature and precipitation, which includes 72.6% (n = 8532) of all cases. The blue quadrant II indicates an increase in precipitation, but a decrease in temperature. This applies to only 2.6% of all cases (n = 305). The orange quadrant III symbolizes negative trends in both parameters. Again, only 2.7% (n = 322) of all cases belong to this class. Finally, the red quadrant IV determines an increase in surface air temperature, but a decrease in precipitation. In 22.1% of the cases (n = 2591), such a combination of trends has been observed. For each quadrant, the median of the observed trends in both climatic parameters is listed in Table 1.

**Table 1:** Median of observed absolute trends (significant at the 10% level) for the climatic parameters mean annual surface air temperature and mean annual total precipitation per direction (quadrant) of change as shown in Fig 3A.

Quadrant	Median of trend in mean annual surface air temperature (K)	Median of trend in mean annual total precipitation (mm)
I	0.787	124.47
II	-0.640	150.78
III	-0.563	-245.99
IV	0.731	-131.48

### Global trends in heat indicators

The previously described long-term trends are overlaid by short-term variability in either climatic parameter, e.g., resulting in extreme events such as intense rainfall or heat spells. Focusing on major indicators for estimating trends in heat, Fig 3B shows the combined shares of (non-)significant (positive and negative) trends in the total number of (non-consecutive) heat days per year, as well as the total number of heat spells per year, and per direction (quadrant) of change. There seems to be a moderate to high association between significant observations, i.e., irrespective of the actual direction or magnitude of either trend, significant trends in the number of heat days per year generally seem to lead to a significant change in the number of heat spells per year (Fig 3B). This seems to apply particularly for the fourth quadrant, which is seen as predominantly at risk of increasing heat extremes and drought due to increasing temperatures and simultaneously declining precipitation.

**Fig 4:**
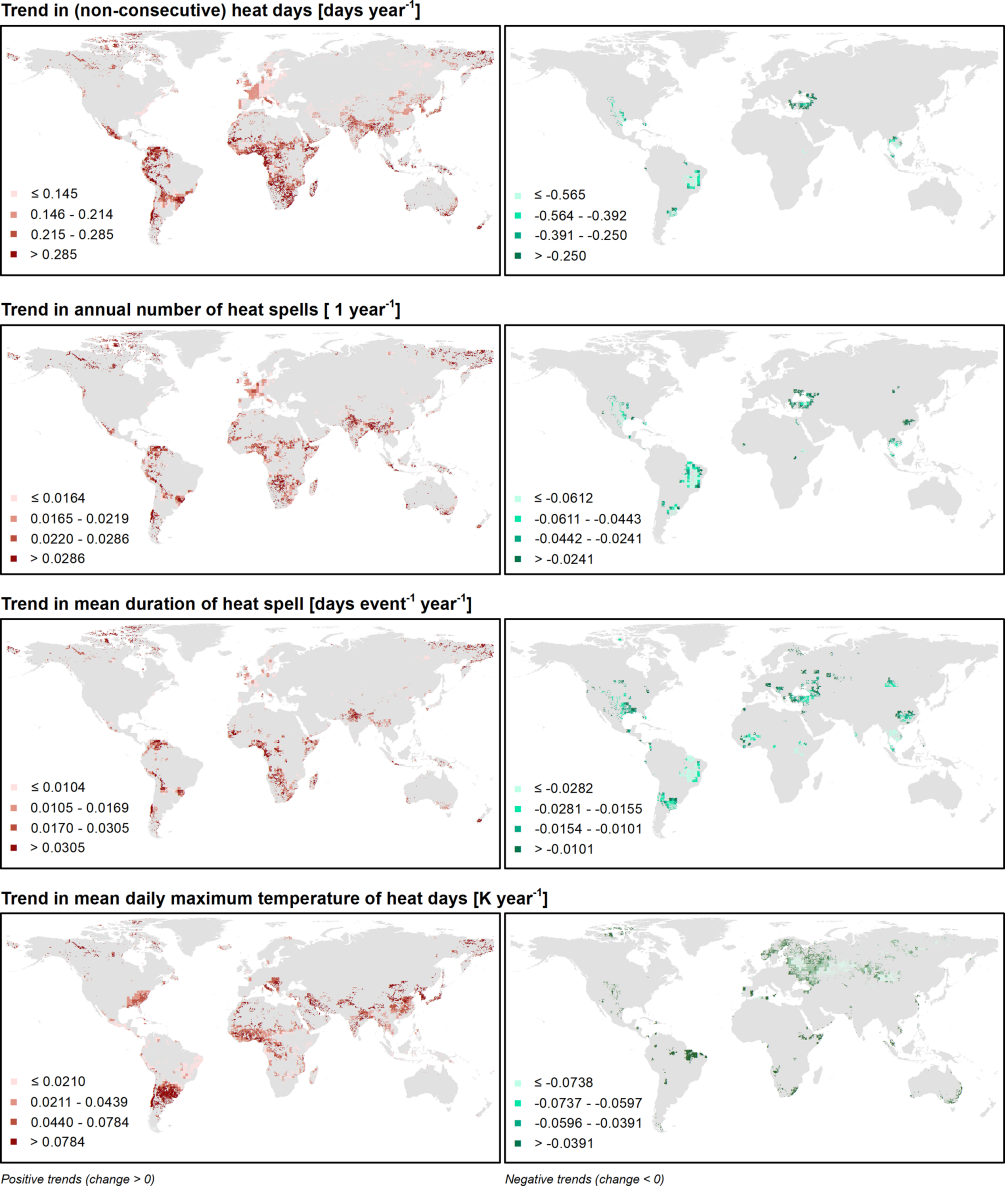
Long-term trends 1901–2000 in the parameters total number of (non-consecutive) heat days per year, total number of heat spells per year, mean duration of heat spells, and mean daily maximum temperature of heat days, as indicators for heat extremes. The left panels show positive trends; the right panels show negative trends (all trends are significant at the 10% level).

Taking also the direction of change into account, a positive (negative) association exists between positive (negative) long-term temperature trends and positive (negative) trends in both the number of heat days and heat spells. For quadrant I, the corresponding phi correlation coefficient, determined using crosstabs statistics, is equal to *φ*_*I*_ = 0.604. For observations in quadrant II, phi is equal to *φ*_*II*_ = 1.000, i.e., positive (negative) long-term trends are matched by corresponding positive (negative) trends in heat indicators. In quadrant III, phi is *φ*_*III*_ = 0.846, and in quadrant IV it is *φ*_*IV*_ = 0.753 (all correlations are significant at the 1% level). Consequently, significant increases of (non-consecutive) heat days per year tend to result in a significantly increasing number of heat spells per year. The global patterns of trends in both heat indicators are shown in Fig 4, which additionally includes the mean duration of heat spells in days and the average maximum daily temperature of a heat day.

However, general long-term trends in mean surface air temperature must not necessarily coincide with trends in heat indicators, e.g., increasing daily maximum temperatures and subsequently heat days or heat spells. Nonetheless, with all trends significant at the 10% level, a spearman correlation reveals that such a coincidence of trends can indeed be observed overall for the quadrants III and IV. For quadrant III, the correlation coefficient (all correlations significant at the 1% level, two-sided) between the trend in mean annual surface air temperature *air* and (i) the total number of (non-consecutive) heat days *h*_*d*_ per year is *r*_*air*−*hd*,*III*_ = −0.371, and (ii) the total number of heat spells per year *w*_*h*_ is *r*_*air*−*wh*,*III*_ = −0.251. For quadrant IV, the respective coefficients are *r*_*air*−*hd*,*IV*_ = 0.335, and *r*_*air*−*wh*,*IV*_ = 0.138.

For quadrants I and II, the associations between the observed trends are weaker. For quadrant I, no significant correlation between the variables *air* and *h*_*d*_ can be found. However, a slight increase in the total number of heat spells per year can be observed, with *r*_*air*−*wh*,*I*_ = 0.116. This may be due to a changing distribution of heat days over a year. For quadrant II, the trend in *h*_*d*_ generally seems to follow the overall long-term trend of decreasing mean surface air temperatures (*r*_*air*−*hd*,*II*_ = −0.296, significant at the 5% level, two-sided). However, the association between the parameters *air* and *w*_*h*_ is contrary to this trend, with *r*_*air*−*wh*,*II*_ = 0.640. This implies that despite the declining total number of (non-consecutive) heat days, the number of heat spells per year increases, likely at the expense of the mean duration of a heat spell. Thus, in comparison to quadrants III and IV, the changes observed in quadrants I and II appear more heterogeneous.

### Integrative perspective on global climate change

So far, we have elaborated on (combined) global patterns of long-term trends and trends in heat indicators. Adaptation to climate change should draw on possibly encompassing information. Consequently, adaptation should regard both long-term trends as well as changes in extremes. This results in comparatively complex information to be assessed and effectively communicated not only to decision-makers, but also to the public. In the following, we suggest an integrative perspective on long-term climate change with a local focus, specifically to facilitate the communication and discussion of climate change and climate change-related risks. This perspective should help answering questions such as “How affected is my community overall compared to …?”, or “Is my city particularly affected by climate change?”

**Fig 5:**
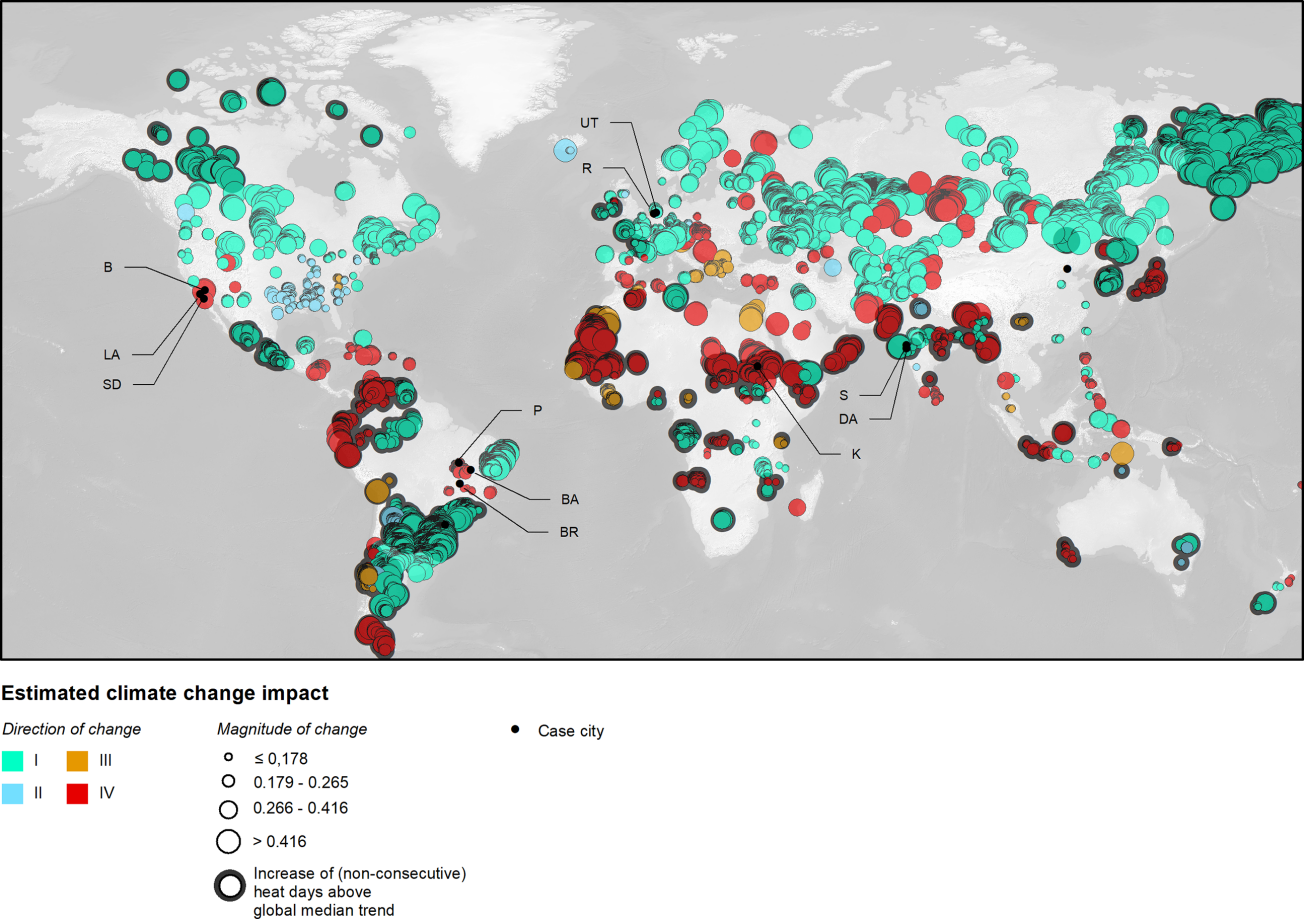
Direction and magnitude $\vec{d}$ of observed relative changes for urban land, with the underlying measures *d*_*air*_ and *d*_*prc*_ significant at the 10% level. The direction of change is indicated by colour, where I equals warmer and wetter; II colder and wetter; III colder and drier; and IV warmer and drier conditions (cf. Fig 3A). The classification of the magnitude of change corresponds to quartiles. Case city abbreviations are as follows: B, Barstow (San Bernardino County, US); BA, Barreiras (Bahia state, Brazil); BR, Brasilia (DF, Brazil); DA, Daman (India); K, Khartoum (Sudan); LA, Los Angeles (Los Angeles county, US); P, Palmas (Tocantins/Brazil); R, Rotterdam (Netherlands); S, Surat (India); UT, Utrecht (Netherlands). Highlighted in black are grid cells where the trend in (non-consecutive) heat days *h*_*d*_ (p < 0.10) exceeds the global median trend in heat days (${\tilde{h}}_{d}=+0.2$ heat days/year).

As described above, we propose the Euclidian distance $\vec{d}$ of the relative trends in mean annual surface air temperature *d*_*air*_ and mean annual total precipitation *d*_*prc*_, respectively, as integrative, quantitative measure for this purpose (Eq 1). Fig 5 shows the global pattern of this measure. To further emphasize the importance of heat as an extreme, Fig 5 also highlights urban land for which an increase in (non-consecutive) heat days above the global median of +0.2 heat days/year has been estimated (p < 0.10).

### Example cases

**Fig 6:**
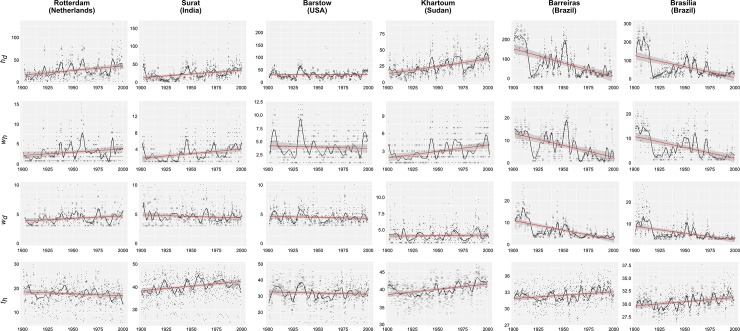
Long-term trends 1901–2000 in heat indicators per case city. The case cities are Rotterdam, Surat, Barstow, Khartoum, Barreiras, and Brasília (from left to right). In the scatterplots, each data point corresponds to the estimated annual value of the respective indicator. To capture the entire urban area, data points are shown for the grid cell at the city’s location and its neighbouring grid cells defined by the first-order Moore neighbourhood. The cross "x" depicts the parameter's annual median value of the selected grid cells. The solid black line corresponds to a locally weighted regression (loess) curve, the red line corresponds to a linear trend model, the shaded area corresponds to the 95% confidence interval of the estimate linear trend. The parameters are as follows: *h*_*d*_, total number of (non-consecutive) heat days per year; *w*_*h*_, total number of heat spells per year; *w*_*d*_, mean duration of a heat spell (in days); *t*_*h*_, mean daily maximum temperature of the heat days of a given year (°C).

Clearly, as discussed earlier and as shown in Fig 3 and Fig 5, increasing precipitation in combination with increasing surface air temperatures is one of two dominant directions of change. Due to the excessive warming and the increase in precipitation in higher latitudes, urban areas located there are mainly affected by this combination of trends. Here, hydrological risk, i.e., risk of flooding, will likely increase for the urban land [4]. As mentioned earlier, this flood risk may be exacerbated in low-lying regions, which are not only affected by riverine flooding, but also coastal flooding due to sea-level rise. For this, the Netherlands is a prime example. Fig 5 indicates that warming and an increase of precipitation is observed for many Dutch cities over the 20^th^ century. National and communal policies anticipate this trend to continue [52]; amongst other measures, adaptation strategies include an increase of retention capacities and the strengthening of coastal defences to avoid riverine and coastal flooding (Table 2). Long experiences with flooding and water management help to devise and implement these strategies. Older cities such as Rotterdam, with nearly 80% of the city below sea level and one of the largest ports in the world, have gained centuries of disaster and water management experience. Moreover, it is the maturity of comparable cities—i.e., their relatively low population growth or possibly stagnating or declining population, in combination with comparatively low rates of spatial expansion and the application of good governance principles [14]—that fosters the implementation of adaptation measures. In this study, it is estimated that in Rotterdam, during the 20^th^ century, the mean annual surface air temperature increased by approximately 3%, and precipitation by about 10%, with *p*_*air*_ < 0.10, however, with *p*_*prc*_ > 0.10. The magnitude of change is estimated for Rotterdam as $\vec{d}=0.111$. This magnitude of change puts Rotterdam in the 5^th^ decile globally, i.e., including all observations of urban land (Table 3). Regarding heat, Rotterdam’s adaptation strategies need to focus on an increase in the overall number of (non-consecutive) heat days and both an increasing number as well as an increasing duration of heat spells (Fig 6), although there is a substantial variability over the 20^th^ century that hampers the assessment of a clear trend. Moreover, although the number of heat days per has increased, there are indications of declining mean daily maximum temperatures of these heat days (Fig 6). This could facilitate heat adaptation strategies (Table 2).

**Table 2:** Adaptation options for climate change-related hazards and risks.

Risk	Hazard	Adaptation option	Explanation
Hydrological	Extreme precipitation, flooding	Urban green areas	Provide flood retention capacity by expanding urban green infrastructure
		Land-use regulations and urban planning	Restrict use of flood-prone areas
		Relocation	Relocate people and assets from flood-prone areas
		Technical flood defences	Strengthen flood defences such as dams
		Building code regulations	Decrease susceptibility and increase flooding resistance of built-up structures
		Manage sewage system	Provide capacities for extreme precipitation events
		Early warning	Implement and/or enhance early warning systems
Climatological/ meteorological	Heatwave	Urban green areas	Provide passive cooling capacities by expanding urban green infrastructure
		Urban blue areas	Provide passive cooling capacities by expanding urban blue infrastructure
		Building code regulations	Adapt buildings to provide shading, improve ventilation
		Accessibility to home cooling options	Provide affordable resource for air conditioning of residential buildings
		Health	Promote individual health and make health care accessible, particularly for the elderly population
		Early warning	Implement and/or enhance early warning systems
	Water scarcity, drought	Increase public awareness	Increase public awareness to limit water waste
		Regulate water use	Regulate and limit water use to prevent water waste and promote increase of use efficiency
		Land-use regulations and urban planning	Manage urban form to limit water waste, e.g., avoid urban sprawl
		Maintain access to livelihoods	Implement solutions for water provisioning amid conditions of scarcity and drought to ensure access to freshwater
		Early warning	Implement and/or enhance early warning systems

**Table 3:** Percentiles of the univariate distribution of the magnitude of change $\vec{d}$ for the global of urban land.

Percentile	$\vec{d}$
10	0.032
20	0.052
30	0.070
40	0.090
50	0.114
60	0.143
70	0.184
80	0.251
90	0.405

For the city of Utrecht, relative changes in surface air temperature and precipitation are higher compared to Rotterdam, with *d*_*air*_ ≈ 6%, and *d*_*prc*_ ≈ 17% (in both cases, p < 0.10). Thus, the magnitude of change for Utrecht is higher with $\vec{d}=0.183$, placing Utrecht globally in the 7^th^ decile (Table 3).

Adaptation challenges for the cities of Surat or Daman, both located at India’s west coast, are similar in principle, with the direction of change being equivalent to Rotterdam or Utrecht, and thus likely facing increasing hydrological risks, i.e., sea-level rise and more frequent flooding, but also heat [53]. For Surat, this is expected despite the comparatively low increases in surface air temperature of about 2% (p < 0.10), and of about 3% in precipitation (p > 0.10). Consequently, on a global scale, Surat might be considered as only slightly affected by climate change with $\vec{d}=0.041$, corresponding to the 2^nd^ decile. However, compared to e.g. Rotterdam, Surat is in a much less matured state of development, thus featuring extraordinary population growth rates. From 1961 to 2001, the population increased tenfold to approximately 2.4 Mio people, with a decadal growth rate of 93% in the period 1981–1991, and 62.4% in the period 1991–2001 [53]; the population is projected to reach 8.5 Mio in 2031. Similarly, the city experienced rapid spatial expansion, from 8.12 km² (1961) to 326.5 km² in 2009 [53]. Unregulated urban growth resulted in urban developments within flood-prone areas with a subsequent restriction of water flow. Implementing adaptation measures and lessons learned from past experiences of flooding—between 1949 and 1979, Surat was flooded approximately once every four years [53]—is substantially hampered by this rapid development. Also regarding heat, Surat faces increased risks, which may severely affect human well-being and human health. Although the average heat spell duration is stagnating, and thus not showing a clear trend, Surat faces an increase of (non-consecutive) heat days, individual heat spells and daily maximum temperatures as risk factors (Fig 6). Also the city of Daman (India), located to the south of Surat on the mouth of the Daman Ganga River, experienced an extraordinary population growth of 69.25% in the decade starting in 2000 [54]. For Daman, increases of surface air temperature and precipitation of 2% and 17% have been estimated over the 20^th^ century (both p < 0.10), resulting in a magnitude of change equal to $\vec{d}=0.169$, thus putting Daman into the 7^th^ decile globally.

As shown in Fig 3A, trends corresponding to increasing precipitation but decreasing surface air temperature are observed substantially less frequent globally. This combination of trends will most likely affect flood risk, e.g., due to more severe extreme precipitation events or coastal flooding due to sea-level rise. The related challenges for managing these hydrological risks are likely similar to the previously explored cases (Table 2).

Trends of decreasing precipitation are predominantly observed in urban areas located in tropical and subtropical climates. Notable hotspots include the Caribbean, the Andean range and the Sahel, which are also hotspots of socioeconomic and ecologic vulnerability. Further affected cities are located in the Mediterranean region, the Middle East, and South-East Asia. In most of these cases, as indicated in Fig 3A, trends of decreasing rainfall are combined with trends of warming. For the affected urban areas, this results in an increasing risk of heatwaves, dry spells, water shortages, water scarcity, or drought. Other impacts include an increased risk of forest fire, desertification, and loss of biodiversity [4]. For example, most of the South-Western United States experiences this combination of trends. Particularly affected are the eastern parts of the Los Angeles county, San Bernardino valley (Barstow), and San Diego, where the combined trends of increasing temperature and decreasing precipitation are statistically significant, and the areas thus becoming drier and warmer over the 20^th^ century (Fig 5A). It is estimated that the surface air temperature in Barstow increased by about 5%, and precipitation decreased by approximately 49% (for both parameters, p < 0.10). This results in $\vec{d}=0.495$, putting Barstow in the 10^th^ decile globally.

Heat trends are difficult to detect for these cities. Neither heat indicator shows a clear trend, but a high variability can be observed (Fig 6). However, the number of (non-consecutive) heat days per year and the number of heat spells per year remained above the estimated linear trends at the end of the 20^th^ century. For this period, also the mean daily maximum temperatures of heat days have been increasing. This coincides with severe drought conditions repeatedly experienced in this region. Starting approximately in the 1990s, large parts of the South-Western United States, and in particular the South of California, started to encounter exceptional drought conditions [55,56]. A most recent drought period started in 2011/2012 and is estimated to represent the worst cumulative rainfall deficit on record, resulting in reduced generation of hydroelectricity [57] and a subsequent boost of CO_2_ emissions due to increasing consumption of natural gas [55], diminished snowpack, and reduced water levels in the Colorado River as well as in Lake Mead and Lake Powell reservoirs [56]. Both reservoirs feed large urban agglomerations, e.g., the rapidly growing Phoenix metropolitan area with an approximate population of 4.5 Mio in 2015, and projected 6.8 Mio in 2040 and 7.7 Mio in 2050 [58]. The sprawled Los Angeles county region [14], with a population of 10.24 Mio (2016) the most populous county in the US [59], is also fed from the Colorado watershed via the Colorado River Aqueduct, and thus possibly at risk of water shortages if conditions are further deteriorating.

A risk of increasing drought is also estimated for North Africa, West Africa and the Sahel (Fig 5). More than 40 percent of Africans live in arid, semi-arid, and dry sub-humid areas. The amount of water available per capita in Africa is already far below the global average and is continuously declining—with annual per capita availability of 4,000 cubic meters compared to a global average of 6,500 cubic meters [60]. Drought is endemic to many African regions and repeated drought cycles killed thousands of people. In addition, the groundwater table is lowered and rainfall is declining in many regions [60]. For example, in Sudan, in line with our findings, changes in climate parameters are summarized by reduced precipitation, warming, solar dimming, increasing evapotranspiration, intensifying aridity and an increasing recurrence of drought events [61]. For Khartoum, the capital of Sudan, relative changes over the 20^th^ century are estimated as *d*_*air*_ = 3.2%, and *d*_*prc*_ = −35% (p < 0.10). With $\vec{d}=0.354$, this puts Khartoum in the 9^th^ decile globally, and thus at comparatively high climate change-related risks. Khartoum also faces an increase in heat; in line with the long-term trend in surface air temperature, the number of heat days per year, as well as the mean maximum daily temperature of heat days, increased significantly over the 20^th^ century. The frequency of heat spells per year increased accordingly (Fig 6).

An increased risk of drought and subsequent aridification and desertification is also estimated for the so-called *Poligono das Secas*, North-Eastern Brazil’s (NEB) Drought Polygon [62], which encompasses several federal states, amongst them the state of Bahia, and which—to a large extent—features semi-arid conditions in the so-called *Sertão* [63,64]. The *Sertão*, as well as NEB as a whole, is repeatedly affected by droughts. For example, the 1997–1999 drought affected about 10 Mio people and more than 1200 municipalities [63]. Typically, and similarly to Sudan, mostly the poor and their livelihoods are affected—and following Kenny, about half of Brazil’s poor live in the region—due to factors such as the loss of livestock and crops, access to water etc. This often leaves migration as the only feasible option for the most vulnerable population [63]. Climate change projections based on a downscaled HadCM3 model for the A1B scenario suggest warming of up to 2° until 2040 and 4° for the whole region in 2071–2100 alongside a reduction of rainfall particularly over the western parts of NEB, which are currently located at the fringe of the semi-arid zone [62,64]. Our findings suggest that this trend can already be observed, e.g., in Barreiras (Bahia, Brazil), or Palmas (Tocantins/Brazil).

In Barreiras, the estimated relative change in precipitation is about -5.1% (p > 0.10), and in mean surface air temperature approximately +2.7% (p < 0.10). The relative changes estimated for the city of Palmas are -11.6% and +1.5%, respectively, with p < 0.10 for both parameters. The corresponding magnitude of change for Barreiras is $\vec{d}=0.059$ (3^rd^ decile), and for Palmas $\vec{d}=0.117$ (6^th^ decile). In line with these findings, also the mean daily maximum temperature of heat days has increased, thus underlining the potentially increasing drought risk and risk of desertification. Our findings indicate overall declining trends in the remaining heat indicators, however, and similarly to Barstow, with observations above the estimated trend in the late 20^th^ century. This also holds true for Brasília, Brazil’s capital region located in the *Distrito Federal* (DF), Brazil’s Federal District. The DF is another example of water scarcity induced by overstraining natural resources due to the intensification of agriculture, urbanization and population growth. In the DF, expansion of intensive agriculture and urban sprawl have caused severe losses of native savanna vegetation (*cerrado*) and put an enormous pressure on the region’s water resources [65–67]. Since the late 1970s, river basins show a dramatic decrease of base flow discharge by 40–70%, presumably due to irrigation [67]. The situation in the DF is pressing, since 94% of the population lives in urban areas, where system capacities for water supply are already close to their limits. Water demand will continue to increase in the near future, with the population projected to grow from currently 2.5 Mio to 3.2 Mio in 2025 [67,68]. However, and contrary to these increasing demands, the relative change in precipitation has been estimated with *d*_*prc*_ = −15.7% over the 20^th^ century (p < 0.10). This is likely putting more stress on the local water supply. The intense use of water for both public supply and agricultural production causes already societal conflicts and environmental problems [69]. Civil unrest related to droughts could already be observed in the NEB [63]. The observed change in mean annual surface air temperature in Brasília is not significant, with *d*_*air*_ = 0.4% (p > 0.10). Nonetheless, due to the substantial decrease in precipitation, Brasília is in the 7^th^ decile globally, with $\vec{d}=0.157$.

### Magnitudes of observed climate change for world regions

Highlighted by selected case cities, we have argued thus far that assessing the direction and magnitude of observed changes in climate parameters is a quick means to identify likely impacts of climate change, related hazards, and risks, thereby facilitating adaptation. We have further demonstrated the application of the proposed integrative measure of climate change, e.g., to classify cases into “risk deciles”. In this regard, actions for adaptation and building of resilience are seen as the more urgent the higher, and thus the more drastic, the estimated magnitude of climate change. The presented “risk deciles” may represent a comparative classification of this scale of change, which varies considerably though as a function of geographic location. Thus, in the following, the magnitude of observed change for the global urban land is aggregated per world region. In so doing, the identification of highly impacted areas at global scale shall be facilitated.

**Fig 7:**
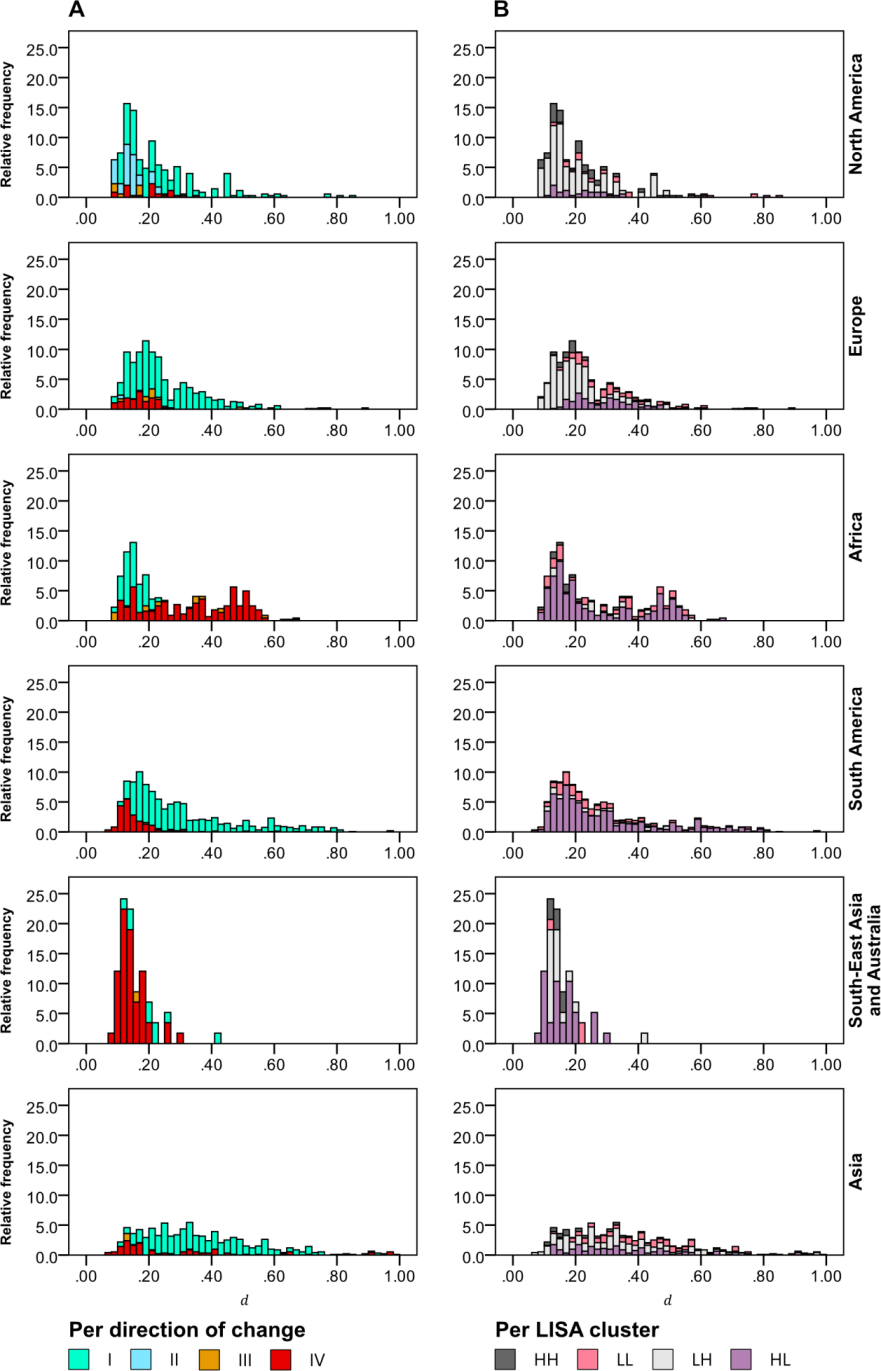
Histograms of the relative frequency (%) of the magnitude of change $\vec{d}$ per world region. (A) Relative frequency as a function of the observed direction of change. (B) Relative frequency as a function of LISA cluster: HH, High-high; LL, Low-low; LH, Low-high; HL, High-low [14]. Cumulative relative frequencies less than 100% indicate values with $\vec{d}>1$ which have been omitted in the figure for clarity.

Fig 7A visualizes the magnitude of change $\vec{d}$ for the global urban land, aggregated on the level of world regions. In addition, the direction of change is considered. Here, the total urban area corresponds to all grid cells of each world region that are a member of one of the four LISA clusters [14], and where the combined trends in both climatic parameters are significant at the 10% level.

It is obvious that the various world regions are differently affected in regard to the predominant direction and the magnitude of change. In principal, Fig 7A replicates the findings depicted in Fig 5, thus showing the dominating trends of climate change over the 20^th^ century per world region. Clearly, warming in conjunction with increasing rainfall is a major direction of change observed in Europe, South America, and Asia, affecting 80.8%, 79.4%, and 77.5% of the exposed total urban area in these regions (S1 Table). The share of urban land exposed to this direction of change is also considerable in North America with 63.2%, and even affects 34.0% of African cases. The corresponding share is lowest in South-East Asia and Australia, with only 15.5%. However, looking at $\vec{d}$, it becomes clear that the associated risks are likely the highest in Europe, North America, South America, and Asia, and considerably lower for African cities, where $\vec{d}$ in regard to this direction of change is comparatively low.

Looking at Fig 7A and S1 Table, it also becomes clear that North America is the only world region where a significant share of urban land is exposed to wetter but colder conditions, affecting 24.2% of the region’s urban space, however, with impacts assessed as likely low. Similarly, as outlined earlier, the third direction of change, i.e., colder but drier conditions, are also only rarely observed. This type of change is mostly observed for African cities, followed by North America and Europe, with a share of 6.8%, 4.0% and 3.7%, respectively.

Finally, warmer and drier conditions are estimated for a substantial share of the global urban land. In North America, this share is lowest with only 8.5%, followed by Europe, South America, and Asia with 14.6%, 19.3%, and 20.8%, respectively. This is in stark contrast to the South-East Asian and Australian region, where warmer and drier conditions, and thus likely increasing risks of drought, are faced by 82.8% of the affected urban land, followed by Africa with 59.2%. However, the observed magnitude of change $\vec{d}$ is considerably higher for many African cities compared to the cases located in South-East Asia/Australia.

### Magnitudes of change for the global urban land

It has earlier been argued that, besides the magnitude of observed change, also the maturity of urban land may affect the implementation of adaptation measures. For example, looking at the estimated magnitude of change $\vec{d}$, Surat may be considered as less affected when compared to Rotterdam or Utrecht. However, adaptation challenges are considerably higher for cities like Surat due to their excessive population growth and urban expansion, which are both indicative for their relative “immaturity”. Consequently, we believe that the state of urban development needs to be considered in impact analysis, since certain stages of the urban development cycle may pose additional barriers for adaptation. Hence, in the following, the magnitude of change $\vec{d}$ is being evaluated in respect to the state of urban land development.

We consider four clusters of bivariate spatial autocorrelation (LISA), that have been interpreted to signify distinct processes or corresponding states of urbanization [14]. A so-called high-low cluster—in the following, HL—has been considered as representative for recent and ongoing urbanization. A low-low cluster (LL) is thought to typify comparatively recent urban spread, i.e., urban sprawl due to suburbanization or peri-urbanization. Both clusters can be considered as less matured states of cities. A third cluster—high-high (in the following, HH)—has also been interpreted to indicate urban spread. However, this HH cluster is seen to characterize comparatively old, large-scale planned city expansions that are typical, e.g., for (Eastern) Europe in the 1960s to 1980s. Finally, a fourth cluster, the low-high cluster (LH), is described to embrace a "final", i.e., matured state of urban development, being characterized by comparatively old age and low variability in spatial extent [14].

When looking at the different states of the affected urban areas as indicated by the share of each LISA cluster per world region (Fig 7B), major differences between the world regions become apparent. In Fig 7B, the magnitude of change $\vec{d}$ is plotted against the relative frequency of the four LISA clusters. Hotspots of ongoing urbanization are marked by the LL and HL clusters [14]. The combined share of both clusters is particularly high in the Global South, i.e., in Asia (50.4%) and South-East Asia/Australia (58.6%), in Africa (86.9%) and South America (89.9%). This is indicative of the ongoing and comparatively rapid urbanization that these regions are experiencing [70]. For example, it has previously been shown that urban areas in Africa are amongst those with the highest magnitudes of climate change, giving rise to a relatively high demand for adaptation. The challenges resulting from the rapid urbanization that these cities are experiencing, as indicated by the corresponding LISA clusters, further increase this demand for sustainable planning, and create needs for additional adaptation due to the increasing number of urban dwellers exposed to climate change. Consequently, a co-management of these two major drivers of global change is highly desirable under these circumstances.

Contrary to that, in Europe, the combined share of the HH and LH clusters is high with 63.5%, compared to Africa with only 13.1% (S2 Table). Both clusters indicate that the affected urban areas can be considered as relatively “mature”, due to factors such as their old founding age, the lack of recent large-scale urban growth, low rates of spatial expansion, and low population growth rates or stagnating or declining populations. Such “mature”, i.e., stable conditions could generally be considered as beneficiary for the implementation of adaptation measures. However, these cities may need to face other vulnerabilities as a consequence of their sociodemographic structure, particularly due to the aging of their population.

## Discussion and conclusions

In this paper, we used the Theil-Sen estimate, a robust regression method often used in climate change research, to identify (significant) absolute long-term trends in mean annual surface air temperature and mean annual total precipitation as possible drivers of long-term stresses. The identified trends, which were found to be in line with earlier studies, were subsequently normalized relative to an early 20^th^ century baseline. The relative changes obtained in so doing were then mapped onto global urban land, and consequently integrated in the form of the Euclidian distance $\vec{d}$, which has been proposed as an integrative, quantitative measure of the magnitude of climate change, i.e., as a measure of the combined observed changes in both climatic parameters. The observed long-term trends were further classified into four quadrants or directions of change, according to the sign of both observed trends.

In this study, heat extremes have furthermore been considered as an immediate and direct impact of climate change at local level. In comparison, hydrological extremes and risks of flooding are substantially more dependent on basin characteristics, i.e., the urban hinterland and rural space, and thus more difficult to assess. Consequently, a focus has been put on heat patterns, which were simultaneously assessed by determining trends in the number of (non-consecutive) heat days and the number of heat spells. Attention was also paid to the mean duration and the average daily maximum temperature of heat spells in order to account for these short-term shocks and their changing patterns as a consequence of climate change. However, trends in hydrological extremes should urgently be revisited in future work.

Firstly, regarding the elicited directions of change, we have shown that warming has been observed as a very likely trend (p < 0.10) for the vast majority of global urban land, i.e., for 94.7% of grid cells that are classified as urban. Looking at the combined trends of warming and very likely trends in mean annual total precipitation (p < 0.10), it can further be concluded that approximately three quarters of global urban land faces trends of increasing precipitation. As shown in Fig 5 and Fig 7A, urban areas affected by increasing temperatures and wetter conditions are mostly located in Northern and Western Europe, North America, Northern and Central Asia, and South America. Warmer and drier conditions are primarily found in urban areas in Central America, Northern Africa and the Sahel, Eastern Europe, and, to some extent, South-East Asia. By additionally considering the observed trends in heat indicators, it has furthermore been shown that these urban areas are also increasingly affected by heat extremes.

Secondly, it is assumed that each observed direction of change is associated with specific climate change-related risks. Wetter conditions are likely associated with increasing hydrological (flood) risks. In combination with warming, this trend may pose additional risks for human health due to the spreading of vector-borne diseases [71]. Drier conditions likely lead to an increasing risk of water scarcity for both humans and ecosystems, and drought. Warming further exacerbates this risk. The associated increase of heat extremes is seen as an additional risk factor.

Thirdly, regarding the proposed integrative measure of the magnitude of climate change $\vec{d}$, it has been argued that $\vec{d}$ is higher when there are higher observed changes in mean annual surface air temperature, or mean annual total precipitation, or both. Consequently, we argue that the impact of climate change, manifested in form of the observed trends in these variables or changes in extreme events, is higher when $\vec{d}$ is higher, and thus, the higher $\vec{d}$, the greater the need for adaptation. In this context, as described above, the elicited directions of change indicate likely risks that need to be considered.

Fourthly, it has been argued that the state of urban development is an important factor in the implementation of adaptation measures, which may be hampered in case of less mature cities due to their ongoing urban expansion both spatially and in terms of population growth [14]. This is especially true for the fast growing (mega)cities in the Global South, as exemplified by the case of Surat, where such urban expansion is not only more rapid, but also less controlled and policy-guided compared to more developed countries.

Finally, in conclusion, it becomes clear that, to a large extent, urban areas in the Global South, particularly Africa, Central and South America, and South-East Asia, are hotspots of climate change as well as hotspots of barriers to adaptation. This is due on the one hand to the prevailing direction of change and the comparatively high magnitudes of observed changes (Fig 7A) as well as, on the other hand, to the wide-spread immature state of urban space (Fig 7B) in conjunction with low economic development and the large amount of vulnerable people [13,15,72].

Moreover, any of the hazards and risks associated with the observed climate trends—in particular heat extremes, water scarcity, and drought—have further implications for the socioeconomic development of cities. For example, threatening the health and well-being of urban dwellers, their food security, as well as the energy and resource security of the urban economy [73], can potentially result in civil unrest or armed conflicts [63,74]. It has also been shown that urban expansion further exacerbates climatological risks in which a scarcity of water is not only a result of a lack of supply due to a deficit of precipitation, but also a consequence of increasing residential, economic and agricultural demand, especially in conditions of warming [73]. Despite urban expansion, water demand is also dependent on urban topology. It has been shown that dispersed, low-density (suburban) housing, typical of urban sprawl, is associated with higher domestic water consumption in comparison to compact, high-density developments [75,76]. In either case, increasing demands for freshwater can ultimately deplete an ecosystem’s ability to provide services and additionally impact human health and regional-to-global migration. This can lead to calls for more sustainable water management [77], but also underlines the need for an effective management of urbanization. Hence, making a case for co-managing urbanization and climate change is particularly needed in the aforementioned areas of the Global South in order to reduce hindrances and barriers to adaptation. Consequently, the assessment of adaptation gaps should also consider such a co-management of driver processes.

Nonetheless, in order to facilitate individual adaptation towards climate change and increase the resilience of urban space, action is also needed to reduce the vulnerability of exposed societal groups. For example, drought adaptation measures in the *Sertão* provide a possible blueprint for addressing drought vulnerabilities in Africa. In this case, water tanks to secure livelihoods were constructed that provided access to freshwater for families and municipalities during prolonged dry spells [62]. Increasing heat stress in the form of prolonged periods of heat or increasing heat days overall, requires that vulnerability to heat, particularly in the elderly population, needs to be addressed. This can be accomplished by adapting cities to warming through residential construction and building codes, reductions of energy consumption, retrofitting of existing systems, and optimization of urban green and blue infrastructure [78–81].

Together, the measures discussed above may ultimately result in synergies and benefits that not only assist in adaptation to climate change impacts, but also improve the quality of life. For example, the expansion of urban green spaces could help to reduce flood risks and heat stress by providing retention as well as cooling capacities (Table 2), but this expansion also provides recreational space and contributes to human health and well-being, and may also positively affect urban biodiversity. Here, the intertwined nature of climate change and public health becomes apparent. Within the context of heat extremes in particular, adaptation strategies (Table 2) clearly seek to reduce the sensitivity of the urban space to climate change. By securing access to livelihoods, adaptation strategies also address the climate change-related risks of water scarcity and drought and, accordingly, bolster public health and human well-being [82–84]. Here, we argue that for a more specific assessment of effects, synergies, and trade-offs, the climate change debate and the ecosystem services community should be firmly linked.

Clearly, the conclusions drawn above rely heavily on the reliability and accuracy of each of the underlying data sources. Here, possible errors arise from stochastic sampling error as a result of heterogeneous data density across the globe, as well as from the interpolation of measurements to gridded surfaces [30]. These errors apply to all climate datasets used. To reduce uncertainty in modelled precipitation, particular care was taken to check for the plausibility of extreme values [31]. The relative error in gridded monthly precipitation is estimated to be between ±7 to 40% of the true mean precipitation in regions in which 5 stations were used for interpolation, and between ±5 to 20% in regions with denser data. If less observations are available for interpolation, the results are less certain. This is the case with results in regions of lower certainty like parts of Northern and Central Africa, South America, Siberia, Central Asia and Australia. However, margin of error also reflects the natural variability in precipitation at a given location, and is thus greater when natural variability is higher [31]. Regarding surface air temperature, it is expected that there is less certainty in summer compared to winter, with the overall error range being comparable to the range of error of a 2- to 4-day numerical weather prediction and still below overall climatological variability [34]. There is, however, considerable uncertainty particularly in the tropics and the southern hemisphere for the period before 1935 [34]. This could explain the observed patterns of relatively sharp decreases of heat days, heat spells and heat spell duration for the Brazilian cases, and thus the rather unexpected linear 20^th^ century trends.

Considering the assessed heat extremes indicators, this study’s focus on urban land requires consideration of the urban heat island effect (UHI). It has been shown that gridded climate data, i.e., air temperatures, can indeed reproduce UHI effects, thus showing elevated temperatures in urban compared to rural grid cells, however, at very high spatial resolutions of 1x1 km [85]. With a resolution of 1/2° x 1/2°, and 2° x 2° respectively, the underlying climate data used for this analysis is considerably coarser. Thus, the UHI effect is not expected to be directly or reliably observed due to the mixing of urban and rural grid cells. Consequently, since heat stress is further exacerbated by the UHI, the estimated parameters may actually be underestimating climate change impacts on cities.

Despite these uncertainties, we nonetheless see the approach used in this study, and the proposed measure of change, to be suitable for at-a-glance comparisons of climate change impacts in localities across the globe. We argue that such an easy means of communicating climate change impacts and risks is a valuable tool for stakeholders, and we particularly hope to foster the exchange of knowledge between cities on a global scale. In this regard, the proposed assessment framework could contribute an additional quantitative scale of reference in order to spark further discussion, e.g., within the 100 Resilient Cities network (http://www.100resilientcities.org/). Our approach also provides important anchor points for future research. Firstly, it highlights urban hotspots of climate change and thus identifies areas of risk prevention and related action, and secondly, it emphasizes the aspects and needs that should be taken into account in a more detailed and more specific climate change analysis at regional or local scales, as well as for subsequent assessments of adaptation gaps.

## Supporting information

S1 Fig Workflow to determine the model variables. The climatic parameters mean annual surface air temperature and mean annual total precipitation have been derived from gridded datasets with monthly values. Both parameters are used to determine the direction of change, as well as the proposed overall magnitude of change in form of the Euclidian distance of the relative change of both parameters over the 20^th^ century in reference to a 1901–1931 baseline. The parameters to assess trends in heat, i.e., total number of (non-consecutive) heat days per year, the total number of heat spells, the mean duration of a heat spell, as well as the annual mean air temperature of heat days, are derived from 6-hourly surface air temperature. Days are classified as (non-)heat days depending on the 90% percentile of the maximum daily temperatures from a 31-day window for the reference years 1970 to 2000.
(TIF)

S1 Table Share of urban area affected (% of total number of grid cells) per direction of change and per world region. The directions indicate the following overall trends: Direction I indicates warmer and wetter, and II colder and wetter conditions. Direction III represents colder and drier, and IV warmer and drier conditions.
(PDF)

S2 Table Share of urban area affected (% of total number of grid cells) per world region and per LISA cluster. From the clusters, most recent urbanization, e.g. according to processes of polarization and (suburban/peri-urban) spread, is indicated by the HL and LL clusters, whose share is particularly high in the Global South, i.e., Africa, South America, and (South-East) Asia [14].
(PDF)
