# Supplementary figures and images for: Integrative assessment of climate change for fast-growing urban areas: Measurement and recommendations for future research
Source: PLoS One. 2017 Dec 12;12(12):e0189451. doi: 10.1371/journal.pone.0189451 (PMC5726725; doi:10.1371/journal.pone.0189451)

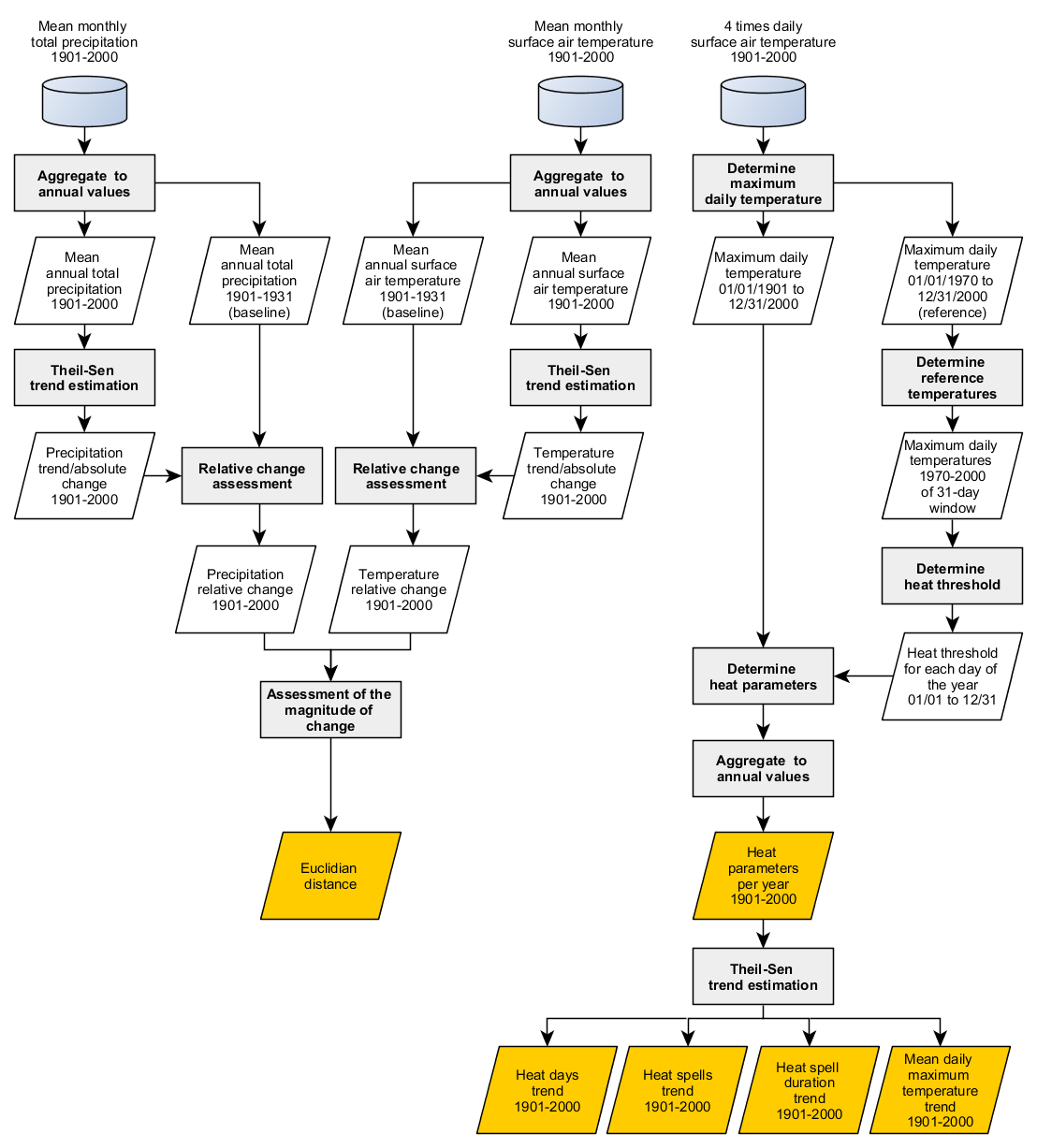

Supplement: S1 Fig — The climatic parameters mean annual surface air temperature and mean annual total precipitation have been derived from gridded datasets with monthly values. Both parameters are used to determine the direction of change, as well as the proposed overall magnitude of change in form of the Euclidian distance of the relative change of both parameters over the 20th century in reference to a 1901–1931 baseline. The parameters to assess trends in heat, i.e., total number of (non-consecutive) heat days per year, the total number of heat spells, the mean duration of a heat spell, as well as the annual mean air temperature of heat days, are derived from 6-hourly surface air temperature. Days are classified as (non-)heat days depending on the 90% percentile of the maximum daily temperatures from a 31-day window for the reference years 1970 to 2000. (TIF) [file pone.0189451.s001.tif]
