# Supplementary material for: Integrative assessment of climate change for fast-growing urban areas: Measurement and recommendations for future research
Source: PLoS One. 2017 Dec 12;12(12):e0189451. doi: 10.1371/journal.pone.0189451 (PMC5726725; doi:10.1371/journal.pone.0189451)
Supplement: S1 Table — The directions indicate the following overall trends: Direction I indicates warmer and wetter, and II colder and wetter conditions. Direction III represents colder and drier, and IV warmer and drier conditions. (PDF) [file pone.0189451.s002.pdf]

S1 Table. Share of urban area affected (% of total number of grid cells) per direction of change and per world region. The directions indicate the following overall trends: Direction I indicates warmer and wetter, and II colder and wetter conditions. Direction III represents colder and drier, and IV warmer and drier conditions.

Region	Direction of change	Share of total (%)
Africa	I	34.0
	II	0.0
	III	6.8
	IV	59.2
Asia	I	77.5
	II	0.2
	III	1.4
	IV	20.8
South America	I	79.4
	II	0.3
	III	1.0
	IV	19.3
North America	I	63.2
	II	24.2
	III	4.0
	IV	8.5
South-East Asia and Australia	I	15.5
	II	0.0
	III	1.7
	IV	82.8
Europe	I	80.8
	II	0.9
	III	3.7
	IV	14.6
