# Supplementary material for: Integrative assessment of climate change for fast-growing urban areas: Measurement and recommendations for future research
Source: PLoS One. 2017 Dec 12;12(12):e0189451. doi: 10.1371/journal.pone.0189451 (PMC5726725; doi:10.1371/journal.pone.0189451)
Supplement: S2 Table — From the clusters, most recent urbanization, e.g. according to processes of polarization and (suburban/peri-urban) spread, is indicated by the HL and LL clusters, whose share is particularly high in the Global South, i.e., Africa, South America, and (South-East) Asia [14]. (PDF) [file pone.0189451.s003.pdf]

S2 Table. Share of urban area affected (% of total number of grid cells) per world region and per LISA cluster. From the clusters, most recent urbanization, e.g. according to processes of polarization and (suburban/peri-urban) spread, is indicated by the HL and LL clusters, whose share is particularly high in the Global South, i.e., Africa, South America, and (South-East) Asia [14].

Region	LISA cluster	Share of total (%)
Africa	HH	5.9
	LL	19.6
	LH	7.2
	HL	67.3
Asia	HH	8.1
	LL	23.8
	LH	41.5
	HL	26.6
South America	HH	1.8
	LL	19.7
	LH	8.3
	HL	70.2
North America	HH	15.7
	LL	8.5
	LH	63.5
	HL	12.3
South-East Asia and Australia	HH	10.3
	LL	5.2
	LH	31.0
	HL	53.4
Europe	HH	7.3
	LL	15.7
	LH	56.2
	HL	20.7

HH, High-high cluster; LL, Low-low cluster; LH, Low-high cluster; HL, High-low cluster.
